# Taste dysfunction as a predictor of depression in schizophrenia: A systematic review and meta-analysis

**DOI:** 10.1371/journal.pone.0300935

**Published:** 2024-03-22

**Authors:** Jia Liu, Shu-Jie Sun, Ye Lu, Xin Ping, Wan Zhang, Lin Pei

**Affiliations:** 1 Hebei University of Chinese Medicine, Shijiazhuang, Hebei, China; 2 Hebei Academy of Chinese Medicine Sciences, Shijiazhuang, Hebei, China; 3 Hebei Key Laboratory of Turbidity, Shijiazhuang, Hebei, China; 4 Renmin University of China, Beijing, China; Public Library of Science, UNITED KINGDOM

## Abstract

**Objective:**

This study aims to investigate the relationship between taste dysfunction and depression among patients with schizophrenia, to achieve early detection of depression in clinical practice.

**Methods:**

Following PRISMA guidance, a comprehensive literature search was conducted globally, covering papers published from 1961 to June 2023. A total of 17 manuscripts were selected through meta-analysis and sensitivity analysis after examining available materials from seven databases to determine the correlation between depression and taste dysfunction.

**Results:**

The comparison of the 17 selected manuscripts revealed that individuals with gustatory dysfunction may be more likely to experience depressive symptoms (SMD, 0.51, 95% CI, 0.08 to 0.93, *p* = 0.02). Depression is associated with taste dysfunction in certain aspects, as indicated by the pleasantness ratings of sucrose solutions (SMD, -0.53, 95% confidence interval [CI] -1.11 to 0.05, *p* = 0.08), gustatory identification ability (SMD, 0.96, 95% CI, 0.03 to 1.89, *p* = 0.04), and the perce*p*tion threshold of sweet taste (MD, 0.80, 95% CI, 0.79 to 0.81, *p* < 0.00001).

**Conclusions:**

Due to variations in the methods, designs, and selection criteria employed in the included studies, it is necessary to establish a feasible framework. Future research using detailed and targeted approaches can provide clearer and more unified conclusions on the relationship between taste dysfunction and depression. Moreover, further high-quality research is needed to obtain clearer conclusions and explore the potential of taste dysfunction as an effective tool for early screening of depression.

**Trial registration:**

This review has been registered in the PROSPERO on April 2022 with the identifier CRD42023400172.

## Introduction

Major depression is a highly prevalent and debilitating condition that significantly impacts personal and public health worldwide, widely recognized as one of the most urgent mental health issues [1]. In the past 30 years, the number of accidents worldwide has increased by nearly 50%, Currently, approximately 280 million people of all ages have been affected [2]. At its worst, depression can lead to suicide, according to the World Health Organization (WHO), more than 700,000 people die per year due to suicide, it is the main cause of global mental and physical disability [3]. It serves as a leading cause of global mental and physical disability and contributes substantially to the overall burden of disease [4]. Objective features, such as perceptual characteristics of the human body and other biological, genetic, and sensory markers, can aid clinicians in identifying individuals with depression. These objective features may facilitate the early detection of depressed patients in clinical settings [5]. Despite the increasing research interest in this field, there is a lack of consistent findings and conclusive evidence regarding the alterations in gustatory function among individuals with depression.

### Taste dysfunction in depressive disorder

Gustatory dysfunction has often been overlooked in patients with depression and those undergoing antidepressant treatment [6]. While numerous studies have investigated the sensory perception of olfactory function in depressed patients, only a few have explored or examined individuals with depression and taste dysfunction [7–24]. In the reviewed literature, some studies have reported a relationship between depression and gustatory function, whereas others suggest no association, leading to controversy. Certain studies have established a reciprocal link between depression and psychophysical cognitive aspects of chemosensation, including gustatory identification ability and hedonic evaluation [25, 26]. Beyond traditional psychophysical measurements, current literature also examines behavioural indicators and analyses their characteristics, duration, and nonverbal communication information [27].

However, the limited number of studies evaluating gustatory function in individuals with depressive disorders, coupled with methodological variations, makes it challenging to interpret and correlate the findings with other research. To gain a comprehensive understanding of the effects of depression on gustatory function, several factors need to be taken into account. These include the assessment of intensity and pleasantness scores of sucrose solutions (hedonic value of the stimulus), the perception threshold of sweet taste, gustatory identification ability, and electrogustometry. The methodology should also consider participant characteristics (e.g., sex, age, causes of depression, disease history and treatment, family history of the disease, and genetic predisposition), the source of taste evaluation (questionnaires, clinical examinations, or self-reports), and the design of different taste concentrations in evaluation methods. The variability in the selection of these variables makes it difficult to reach a consensus regarding the role of depression in gustatory function [28].

### Measurement of taste dysfunction

It is evident that adopting a less biased approach in research evaluation is crucial. Additionally, recording the patient’s medical history or conducting a simple oral examination before initiating clinical examinations is important [29]. Gustatory function can be assessed at various levels [30], including the ability to recognize and differentiate different types of taste. Recognition memory, which assesses the ability to recognize previously encountered flavours, is a feature that can be evaluated under normal and pathological conditions. Oral “taste tablets” have also been proposed as a soluble material developed specifically for gustatory function testing systems [31, 32].

Evaluating changes or impairments in pleasantness, hedonic value, and gustatory identification ability can provide insights into different mental illnesses. Common clinical testing methods [33] are available for assessing different aspects of gustatory function, such as chemogustometry and electrogustometry. Chemogustometry typically involves administering small amounts of taste substances, drug sprays, or filter papers, which are then ingested by the subjects who provide scores. The tongue is rinsed with deionised water between samples to eliminate residual taste. The final score represents the sensitivity of taste perception. Electrogustometry, on the other hand, is a standardised device for studying human taste perception [34]. It assesses thresholds by applying low-intensity current continuously over a specific duration. A lower threshold indicates greater taste sensitivity in the subject [35]. Tests can be categorised as whole-mouth tests or regional tests, depending on the oral testing areas being evaluated. Different gustatory function testing methods can be employed based on the specific objectives of clinical trials, including perception threshold of sweet taste, taste identification, and intensity tests. The measurements of gustatory function utilised in the reviewed literature are presented in Table 3.

### Cause of taste dysfunction

Several factors can harm gustatory function, impacting both physical and mental health. Unhealthy lifestyle habits, diseases, medications, zinc deficiency, oral hygiene, and infections are among the factors that can adversely affect gustatory function [36]. Furthermore, normal ageing can also have an impact on gustatory function. The most common reasons for taste dysfunction include medication (21.7%), zinc deficiency (14.5%), and various mental and physical diseases (7.4% and 6.4%) [37, 38]. Table 1 presents the reasons discussed in the relevant literature.

**Table 1:** Mechanistic cause of taste dysfunction.

Disturbance	Category	Cases
Transport problem	Receptors unable to respond to external stimuli	Oral candidiasis [39], Salivary dysfunction
Perceived impairment	Gustatory sensor malfunction	Radiotherapy and chemotherapy [40], Burn, Trauma [41], Nasal and paranasal sinus diseases [42], and Periodontal disease [42]
Neuronal damage	Injury of cranial nerves or spinal cord nerve, central nervous system	Tongue surgery [42], Neoplasm, Brain tumour, Cerebrovascular accident [42], Burning mouth Syndrome [41, 43, 44], Metabolic disorders [20]
Others	Infection	Upper respiratory infection [41, 42], Initial symptoms of COVID-19 [45]

### Brain regions involved in depressive disorder

The cortical-subcortical circuit is the most common functional association observed in depressed patients [46]. Previous findings have identified regional grey matter variations in the frontal lobe, putamen, parietal lobe, caudate, thalamus, pallidum, and temporal lobes [47–49]. Abnormal brain activity has been observed in the prefrontal cortex, occipital lobe [50], temporal grey matter, caudate [51], and putamen [52]. These various brain regions are interconnected and form complex brain networks, with the putamen, frontal lobe, thalamus, parietal lobe, and hippocampus serving as key hubs within these circuits [53–55]. It is well established that individuals with depression exhibit cognitive deficits across multiple domains [56] and have been shown to experience hippocampal atrophy [57]. The hippocampus, a pivotal limbic system component, is extensively connected with the prefrontal cortex, amygdala, anterior thalamic nuclei, basal ganglia, and hypothalamus [58, 59]. Furthermore, the amygdala, traditionally recognised for its role in associating negative emotional stimuli with contextual cues, also plays a similar role in processing rewarding stimuli [60, 61]. Certain neuropeptide systems in the hypothalamus are known to influence the rewarding responses to addictive substances [62–64].

### Brain regions involved in taste dysfunction

In humans, taste pathways project from the nucleus of the solitary tract directly to the taste thalamus. The taste cortex, located in the anterior insula, provides separate and integrated representations of food temperature, taste, and texture in the oral cavity [65]. The orbitofrontal cortex is a key brain region involved in taste processing [66, 67]. The primary taste cortex in the anterior (granular) insula specifically encodes taste intensity [68, 69]. On the other hand, the orbitofrontal cortex and anterior cingulate cortex are responsible for encoding the reward value of taste, as activations in these regions are associated with the subjective pleasantness of taste experiences [70]. Neurons in the orbitofrontal cortex respond differently to the four classic taste qualities: sweet, salty, bitter, and sour [71]. They can also be stimulated by pleasurable tastes (e.g., sucrose or glucose) or aversive taste stimuli such as quinine or sodium chloride [72]. Certain neurons in the ventral striatum and the head of the caudate nucleus regions respond to the taste, flavour, and/or sight of food [67, 73]. The amygdala and hypothalamus are also involved in taste processing. The human amygdala is particularly responsive to the taste of glucose and contributes to hedonic judgments of taste [5, 74]. Additionally, the hypothalamus plays a role in integrating taste reward signals with hunger and satiety signals [67].

## Methods

### Protocol and registration

This integrated review on the Prospective Register of Systematic Reviews (PROSPERO) was registered on April 2022 (CRD42023400172). The review and meta-analyses were conducted in accordance with a predetermined protocol and adhered to the Preferred Reporting Items for Systematic Reviews and Meta-Analyses (PRISMA) statement.

### Literature search strategy

The literature search was conducted following PRISMA guidance. Seven online databases were utilised: PubMed, Ovid Medline, Embase, Cochrane Library, Web of Science, EBSCO Foreign Language Periodicals Database Analysis, and China National Knowledge Infrastructure [CNKI]. The primary search terms employed were: “Taste dysfunction”, “Gustatory dysfunction”, “Taste disorder”, “Sense organ disorder”, “Dysgeusia”, “Dysgustatory”, “Gustatory function”, “Gustation”, “Taste”, “Taste Sense”. “Depression”, “Depressive disorder”, “Major depression”, “Depressive Disorder, Major”, “Depressive Symptoms”, “Depression, Involutional”, “Depression, Neurotic”, “Symptoms, Depressive”, “Depressed disorder”, “Depressive Syndrome”, “Unipolar Depression” and their associated terms. No date filters were adopted, those associating with humans, and not those with the core term “Parkinson’s disease”.

### Study selection

The titles and abstracts of all papers were screened by two study team members (Jia Liu and Shu-Jie Sun) for data extraction and synthesis. Full-text articles deemed eligible for review were obtained and thoroughly read to determine final eligibility. A standardised data extraction tool was created, including research details (author, year, country), participant information, depression measurement methods, gustatory function assessment utilised, factors contributing to differences, and potential confounders. All the findings were derived from retrospective studies, and the data synthesis focused on the relationship between gustatory function and mental health outcomes.

**Fig 1:**
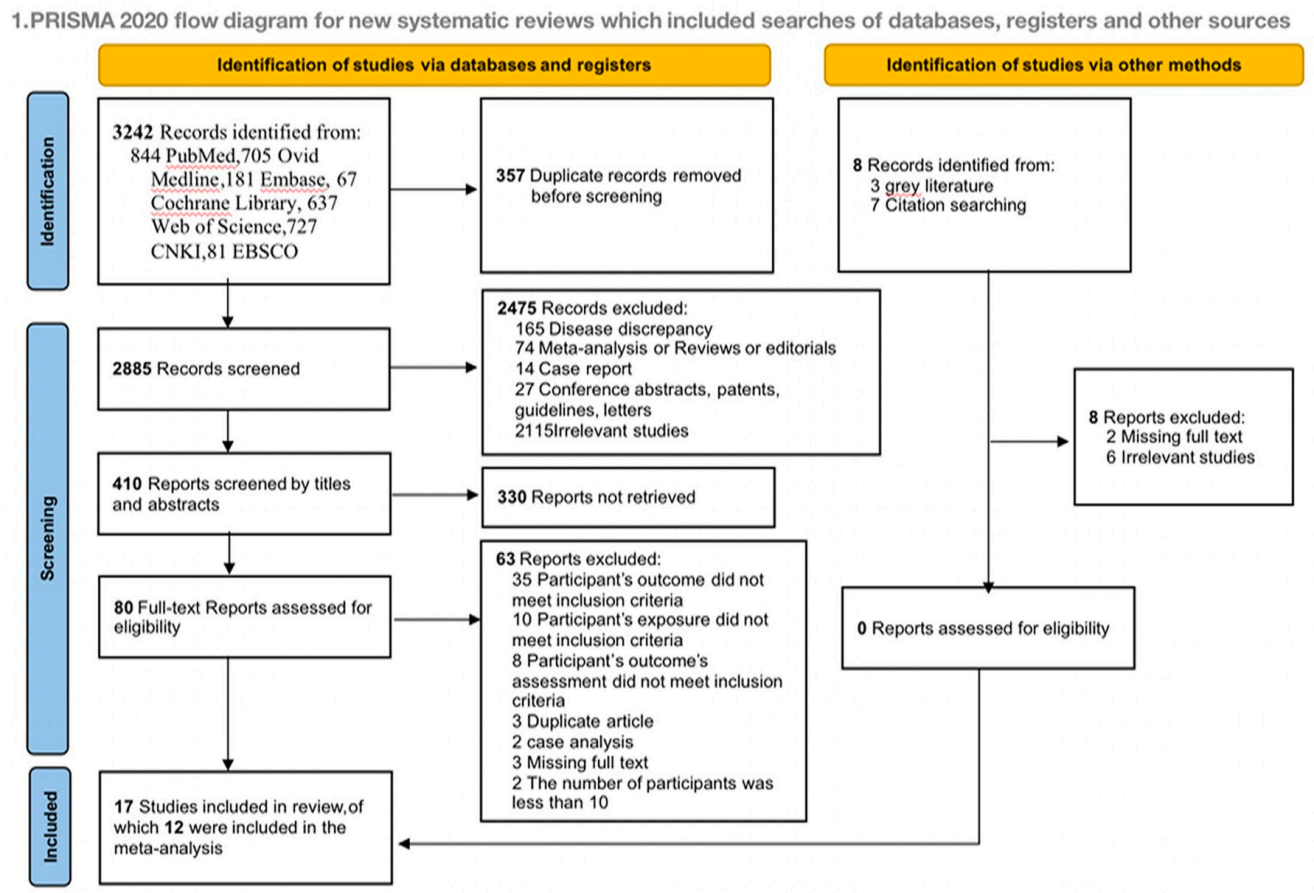
PRISMA flow diagram. PRISMA (Preferred Reporting Items for Systematic Reviews and Meta-Analyses) 2020 flow diagram for new integrated reviews, which included searches of databases, registers and other sources. Latest search date: June 15, 2023.

A total of 3,242 articles were identified through the search process. Additionally, 3 articles were discovered by searching grey literature (https://ntrl.ntis.gov/NTRL/), and 7 articles were found by checking the reference lists of previous integrated reviews. These 3,242 research articles were recorded in EndNote^™^ Basic, and duplicates were identified and removed, resulting in 2,475 unique manuscripts. The titles of these manuscripts were reviewed, leading to a selection of 80 full manuscripts for further evaluation. These 80 articles were obtained and thoroughly read to determine their final eligibility. Based on the full-text review, an additional 63 articles were excluded, leaving 17 articles that met the eligibility criteria. A flowchart illustrating the research selection process is provided in Fig 1. Both authors discussed and resolved any disagreements, reaching a consensus based on the inclusion/exclusion criteria. The references of the reviewed manuscripts were also examined to identify any additional relevant research.

### Inclusion and exclusion criteria

To be included in the initial depression aspect of the research, patients were required to have a documented diagnosis of depression by a healthcare professional or through the use of a validated instrument for assessing depression symptoms. The studies had to provide relevant data on quantitative gustatory function in both depressed patients and non-depressed controls. Articles were excluded if they relied solely on patient self-report for evaluating depression or provided only subjective information on gustatory function. For inclusion in the primary taste dysfunction aspect of the research, patients need to have confirmed taste dysfunction, as determined by either patient-reported symptoms or objective assessment methods, with no restrictions on the underlying causes of taste dysfunction. Each included study was required to report assessed depressive symptoms using a validated depression instrument. Reviews and individual case reports were excluded from the analyses.

P (Patient): Outpatients or inpatients in medical institutions.
I (Exposure): Patients with depression or abnormal taste.
C (control): People without depression or those without abnormal taste. The definition and diagnostic criteria of diseases are basically consistent with the standards applied in the original research.
O (Result): Based on the original study, professional medical personnel used standards to diagnose and record the disease, or evaluated symptoms by using effective tools.
S (Research Design): Case Control Study and Randomized Controlled Study.

### Quality assessment

The methodological quality of each study included in this integrated review was assessed using a modified Newcastle-Ottawa Scale, a validated tool for non-randomised studies in meta-analyses. Initially, 18 articles met the inclusion criteria, while 1 article did not [75]. The study conducted by W. Sperling in 2010 did not meet the quality standards due to the absence of precise measurements, inadequate control group utilisation, and unresolved confounding factors. The remaining 17 studies met the criteria and satisfactorily addressed all nine questions outlined in the Newcastle-Ottawa Scale (NOS), as shown in Table 2. One study conducted by Amsterdam et al. [76] did not explicitly state how they addressed the confounding factors. However, this study was still included in the review as the undisclosed confounders did not significantly impact the applicability of the manuscript, and all other key questions were addressed satisfactorily.

**Table 2:** Quality evaluation on research.

Study source	Selection				Comparability	Exposure			Total score
	Adequate definition of cases	Represent-ativeness of the cases	Selection of controls	Definition of controls	Control for important factors	Ascertainment of exposure	Same method of ascertainment for cases and controls	Non-Res-ponse rate	
Mikhail et al, 2021 (Egypt)	1	1	1	1	2	1	0	0	7
Arrondo et al, 2015 (UK)	1	1	1	1	2	1	1	1	9
Swiecicki et al, 2015 (Poland)	1	1	1	1	2	1	1	1	9
Naudin et al, 2015 (France)	1	1	1	1	2	1	1	0	8
Dichter et al, 2010 (UK)	1	1	1	1	2	1	0	0	7
Swiecicki et al, 2009 (Poland)	1	1	1	1	2	1	1	1	9
Berlin et al, 1998 (France)	1	1	0	1	2	1	1	1	8
Steiner et al, 1993 (Israel)	1	1	1	1	0	1	1	0	6
Amsterdam et al, 1987 (US)	1	1	0	1	1	1	1	1	7
Miller & Naylor, 1989 (UK)	1	1	0	0	0	1	0	1	4
Chen et al, 2021 (Germany)	1	1	1	1	2	1	1	0	8
Carmo Carvalho et al, 2019 (Brazil)	1	1	1	1	1	1	1	0	7
Braud &Boucher, 2016 (France)	1	1	0	0	0	1	0	1	4
Davies et al, 2015 (UK)	1	0	1	0	1	1	1	1	6
Brandt et al, 2008 (Germany)	1	1	1	0	1	1	1	1	7
Heckmann et al, 2005 (Germany)	1	1	1	0	2	1	1	1	8
Deems et al, 1996 (US)	1	1	0	0	0	1	0	1	4

Annotation: The research quality was evaluated based on the Newcastle-Ottawa Quality evaluation scale for cohort research. This scale awards a maximum of 9 points to every research: 4 for selection, 2 for comparability, and 3 for evaluation of results (for cohort research). 1 = “Yes”,0 = “No”, “Unable to decide”, or “Not available”.

### Rating the body of evidence

The outcome indicators included in the randomized trial study were scored on the quality of evidence using the GRADE system (GRADE profiler, version 3.6.1). Randomized controlled trials were first set as high-level evidence, and then downgraded outcome indicators from five aspects, namely bias risk, inconsistency, indirectness, imprecision, and publication bias. If all aspects are not downgraded, it is considered high-level evidence; One aspect of downgrade is moderate evidence; Downgrading is considered low-level evidence in two aspects; The downgrade of three or more regions is evidence of a very low level.

### Data extraction and statistical analysis

Two authors (JL and SJS) extracted data from studies that met the inclusion criteria. The extracted data included demographic characteristics, sample sizes of cases and controls, means and standard errors of depression or gustatory information, and associations and conclusions regarding the relationship between taste dysfunction and depression. The methodology used to measure gustatory function was recorded. If two or more studies reported gustatory information using a validated scale in depressed populations, pooled sample sizes, means, and standard errors were calculated. Similarly, pooled sample sizes, means, and standard errors were calculated for patients with taste dysfunction if two or more studies reported depression information using a validated scale. The heterogeneity across studies was assessed using the *I*^*2*^ statistic. If significant heterogeneity was detected, a random effects model with a corresponding *p* value was employed. If non-significant heterogeneity was found, a fixed effects model with a corresponding *p* value was used. Sensitivity analysis was conducted using a leave-one-out analysis [77] to assess the impact of each individual study. Statistical heterogeneity among studies was evaluated using the *Q* and *I*^*2*^ statistics. For the *Q* statistic, a significance level of *p* < 0.05 was considered statistically significant, while *I*^*2*^ values of 25%, 50%, and 75% were used as cutoff points for low, moderate, and high levels of heterogeneity [78]. Potential publication bias was assessed using Egger’s test. A *p* value of < 0.05 was considered statistically significant for all tests. Most analyses were performed using Review Manager (RevMan 5.4.1) [79], and sensitivity analysis was conducted using STATA/MP statistical software.

### Evaluation of the risk of bias in included studies

**Fig 2:**
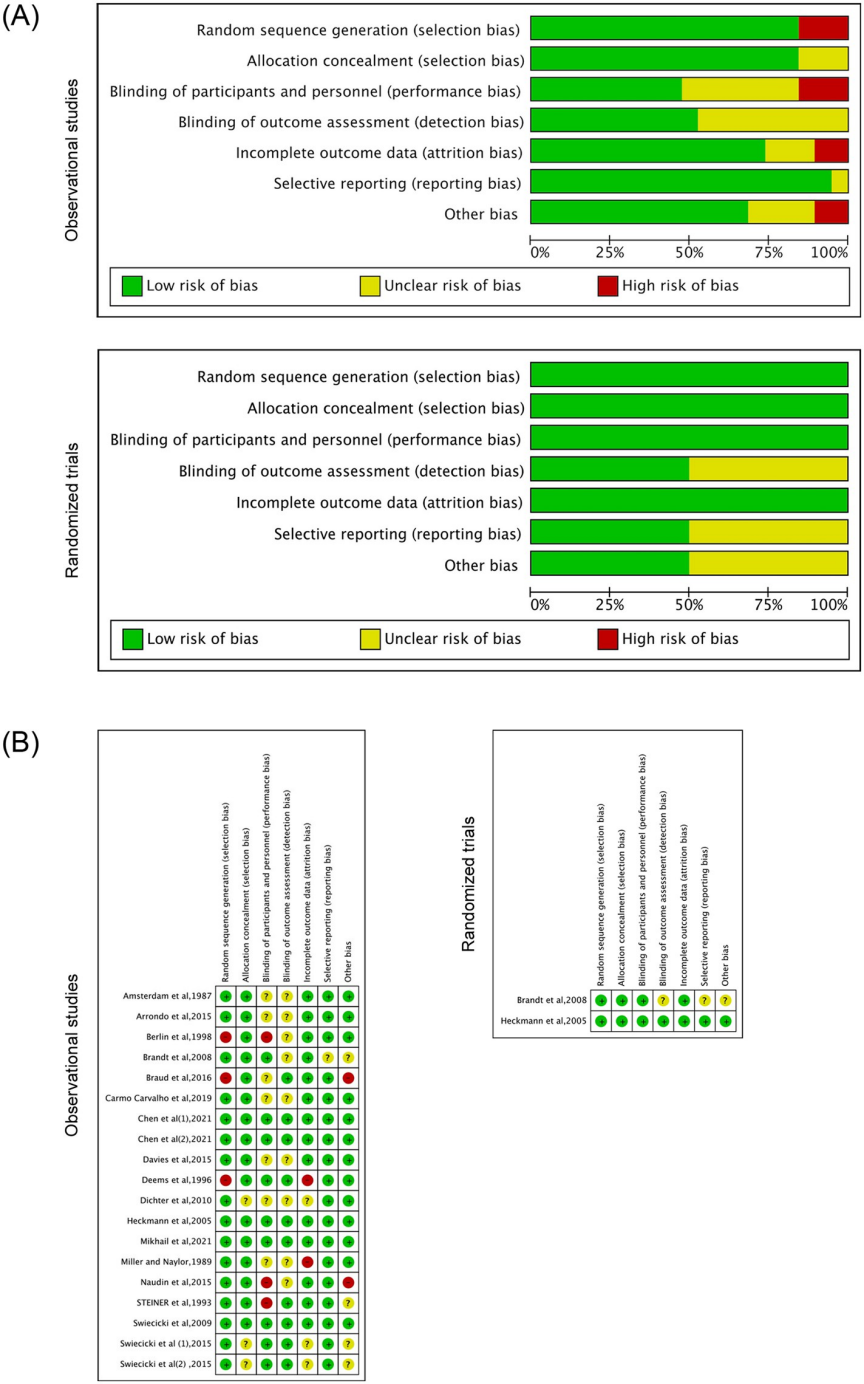
Quality evaluation. A: Every risk of bias domain is shown as a percentage across all included research (observational studies and randomized trials). B: Every risk of bias domain for every included research (observational studies and randomized trials).

Two review authors assessed the risk of bias for each trial using the Cochrane Handbook for Systematic Reviews of Interventions [80] as the standard. Any disagreements were resolved through discussion or by involving a third assessor. The Cochrane Collaboration’s risk of bias tool was utilised in Review Manager (RevMan 5.4.1) software to evaluate the quality of the included studies in our systematic review. The tool consists of six assessment items that address different aspects, including random sequence generation and allocation concealment (selection bias), blinding of participants and personnel (performance bias), blinding of outcome assessment (detection bias), incomplete outcome data (attrition bias), selective reporting (reporting bias), and other potential sources of bias. Each item was assessed as having a low, high, or unclear risk of bias. Fig 2 provides an overview of the risk of bias across the included trials. However, due to inadequate reporting, the overall risk of bias was unclear.

## Results

### Search outcomes

There were 3,242 articles retrieved in the literature search. After removing duplicates, a screening of 410 titles and abstracts was conducted based on the inclusion/exclusion criteria. Of these, 330 articles were excluded, and 80 were subjected to a full-text review. Ultimately, based on Fig 1, the integrated review and meta-analysis excluded 17 articles.

### Risk of bias and quality evaluation

The outcomes of the risk of bias evaluation are summarized in Table 2 and Fig 2. Most studies exhibited a low risk of bias in the randomization process. However, three studies were deemed to have some concerns due to insufficient data regarding randomization. Considering the case-gathering process, blinding of participants was not feasible, leading to several concerns in three studies concerning deviations from the expected interventions. Four studies were classified as “high risk” in terms of outcome assessment due to incomplete result information.

Additionally, one study raised concerns about the consistency of the intervention approach, another study had a significant gender imbalance [44], and one study exclusively included participants over 65 years old [81]. The potential impact of age on the results could not be ruled out, resulting in a “high risk” judgment for selecting the reported outcome. Among the studies included in the meta-analysis, eight studies (47%) had a low overall bias, three studies (18%) had several concerns, and six studies (35%) exhibited a high bias.

### Study characteristics

A total of 843 participants were included in the 17 studies, with 486 patients assigned to the depressed group and 357 to the control group. The publication dates of the included studies spanned from 1987 to 2021. These 17 studies were conducted in eight different countries, with the largest number of studies conducted in Europe (12 studies: 4 from the UK, 2 from Poland, 3 from France, and 3 from Germany). 2 studies were conducted in the United States, while one study each was conducted in Asia (Israel), South America (Brazil), and Africa (Egypt). Among these studies, 10 focused on gustatory dysfunction in depressive disorders, while 7 examined depression in patients with taste dysfunction. Table 3 provides an overview of the study features, presenting a concise summary of the key aspects related to comparing gustatory function assessment and depression measures. To ensure a proper comparison and minimise bias, studies that employed the same gustatory function test were collected.

**Table 3:** Basic characteristics of studies.

Studies source	N^a^	Age Mean y. (SD)	Gender(F/M)	Depression measure	Methods for assessing gustatory function	Thres-hold	Intensity	Identifi-cation	significant difference	Aspect of difference	Confounders
Mikhail et al, 2021 (Egypt)	20	38.6 (7.6)	22/8	NR	Taste identification tests(filter paper disc method)		x		Yes	Threshold,Identifica-tion	None
Arrondo et al, 2015 (UK)	56	35.6 (8.8)	56/0	BDI,HRSD	Taste sensory evaluations	x		x	None		None
Swiecicki et al, 2015 (Poland)	72	36.3 (2.2^b)^, 36.8 (2.1^c)^	51/21	DSM-IV,HAM-D,BDI	EGM,Taste identification tests(filter paper disc method)	x			None		Age,Sex,BMI,Smoking,Education
Naudin et al ,2015 (France)	44	64.9 (11.2)	32/12	MADRS	Taste identification tests,Hedonic response tests,Taste intensity evaluations	x			Yes	Identification,Intensity(Salty and sour) Pleasantness(Salt and acid)	Medication,Age,Smoking
Dichter et al, 2010 (UK)	31	39.07 (10.4)	15/16	DSM-IV,HAM-D	Gustatory detection threshold tests	x		x	None		None
Swiecicki et al, 2009 (Poland)	50	35.7 (2.3)	27 /13	HAM-D,BDI	EGM,Taste identification tests(filter paper disc methods)				None		Smoking
Berlin et al, 1998 (France)	40	47 (18)	26/14	MADRS	Gustatory detection threshold tests, Hedonic response tests			x	Yes	Threshold, Intensity and Pleasantness	Age,BMI
Steiner et al, 1993 (Israel)	37	52.8	20/17	DSM-111-R	Hedonic response tests,Taste intensity evaluations	x			Yes	Identification,Intensity(Sweet)	None

Abbreviations: F female, M male, NR not report, x none, BDI Beck depression inventory, HRSD Hospital anxiety and depression scale, DSM-IV Diagnostic and statistical manual of mental disorders four edition, HAM-D Hamilton depression rating scale (HAM-D)21, MADRS Montgomery and asberg depression rating scale, DSM-111-R Diagnostic and statistical manual of mental disorders third edition modified, DSM-II Diagnostic and statistical manual of mental disorders two edition, CIS-R clinical interview schedule–revised, SRQ-20 Self-Reporting questionaire 20, EGM Electrogustometry.

^a^ N presented for the number of participants included in analyses.

^b^ Sad depressed,

^c^ non-Sad depressed,

^d^ Gustatory dysfunction,

^e^ Mixed gustatory and olfactory dysfunctions.

### Synthesis without meta-analysis in the literature

All results are presented for continuous variables due to a lack of available data on dichotomous variables. In the case of five studies [27, 41, 43, 76, 82], they were not included in the meta-analysis because the effect size estimates were incomplete.

In one study [27], video recording of facial expressive features and duration was used to assess gustatory identification ability and intensity and pleasantness ratings of sucrose solutions. The results clearly indicate that while depressed patients were generally able to recognise and evaluate taste stimuli equally and produce similar mental and physical responses compared to the control group, significant variations were observed in the duration of the sweetness response between the two groups, and the facial expressions were markedly different. These findings suggest that the modulating effect of depression on hedonic evaluation may be attributed to nonverbal responses rather than psychophysical measures.

Another study [41] suggested that depressed patients often complain about unpleasant tastes. However, upon comparing various factors related to this symptom, including sex, age, menopausal status, a previous adequate trial of antidepressants, and a family history of depressive illness, no significant diversity was detected. The study also mentions that taste disorders in hospitalised depressed patients seem to improve with recovery from depression, although this observation is based on individual doctors’ experiences.

In a different study [82], a natural logarithm change was utilised to estimate the pleasantness marks of intensity and pleasantness ratings of sucrose solutions. The results revealed no significant diversity between the non-depressed and depressed groups in hedonic valuation. These findings suggest that depression may impair cognitive evaluation of pleasantness stimuli while preserving more basic pleasantness responsiveness. The conclusion proposes the bold assumption that cognitive interventions should focus not on feelings of pleasant stimuli but rather on feelings of frustration.

Another study [76] found that taste intensity and pleasure measurements were altered in some depressed patients compared to controls. Depressed patients exhibited higher recognition thresholds for sucrose than controls, while there were no significant differences in pleasantness ratings. A more specific conclusion has been suggested by one study [43] that, notably, gustatory function changes were greatly related to excess psychiatric symptoms. Specifically, depressive symptom marks were greatly related to metallic or acidic taste.

### Integrated review and meta-analysis in the literature

Out of the 17 studies reviewed, 12 included a meta-analysis step. Among the six studies focusing on depressed patients, various assessments of gustatory function were utilised. Specifically, four studies incorporated pleasantness ratings of sucrose solutions, three studies employed taste identification tests, two studies utilised electrogustometry [EMG], and the remaining two studies used sweet threshold taste tests. It is worth noting that one study employed the test-retest reliability of gustatory function assessment [83]. In this case, subjects underwent two tests at a 2-week interval, and the results demonstrated satisfactory reliability. These represent the most common and comprehensive gustatory function tests.

The current study’s depression diagnosis was based on the DSM-IV standard in ten studies. Among these, three studies [84–86] repeatedly assessed depression, while the remaining studies evaluated depression at baseline. Regarding the six studies on taste disorders, different measures of depression were employed: 4 studies used Beck depression inventory [BDI], one study used SQF-20, and one study used HAD-D. These rating scales are widely recognised and commonly used for depression testing.

#### Taste dysfunction in depressed compared to the control group

**Fig 3:**
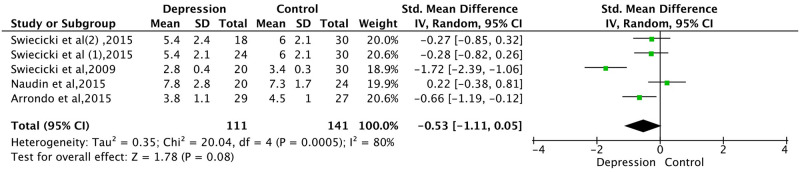
Forest plot of variation in gustatory function based on the pleasantness ratings of sucrose solutions (hedonic valuation). CI, confidence interval; SD, standard deviation; SMD, standardised mean difference. In Swiecicki’s study, data was isolated based on depression only, and winter depression was calculated with mean and standard deviation. Hence, their raw data was adopted; Swiecicki(1) represents depression data, and Swiecicki(2) refers to winter depression data.

Four research studies included a total of 222 participants to compare hedonic evaluations. Fig 3 revealed that controls had slightly better sweet taste recognition ability than depressed individuals, although the difference was not statistically significant (*p* = 0.08, 95% CI, -0.53 [-1.11, 0.05]). However, there was significantly high heterogeneity among the studies (*p* = 0.0005, *I²* = 80%), and due to inconsistent testing methods, we employed standardised mean difference (SMD) merging. The sensitivity analysis indicated that no single study significantly influenced the overall outcome, indicating the overall outcome’s good stability.

**Fig 4:**
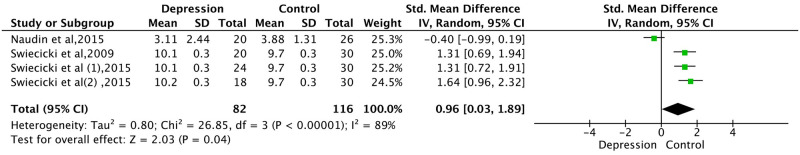
Forest plot of variations in gustatory function based on the taste identification tests. In Swiecicki’s study, data was isolated based on depression only, and winter depression was calculated with mean and standard deviation. Hence, their raw data was adopted; Swiecicki(1) means depression data and Swiecicki(2) means winter depression data.

Three studies involving a total of 168 participants were included to compare gustatory identification ability between depressed patients and non-depressed controls. Fig 4 presents the results of a meta-analysis on taste recognition between the two groups, indicating that non-depressed patients exhibit better taste recognition abilities than depressive patients, with statistically significant differences (*p* = 0.04, 95% CI: 0.96 [0.03, 1.89]). However, there is a high level of heterogeneity among the four studies (*p* < 0.00001, *I²* = 89%).

**Fig 5:**
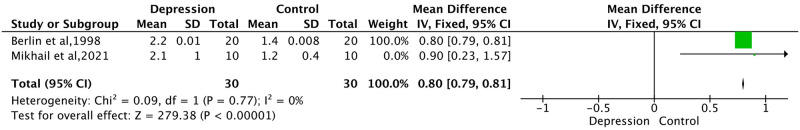
Forest plot of variations in gustatory function based on the perception threshold of sweet taste.

As shown in Fig 5, two studies involving a total of 60 participants reported the perception threshold of sweet taste tests. The analysis results revealed a significant difference, indicating that depressed patients had a significantly lower ability to identify sweet tastes than non-depressed patients (*p* < 0.00001, 95% CI: 0.80 [0.79, 0.81]). Furthermore, the testing methods across the studies were consistent, and no heterogeneity was observed (*p* = 0.77, *I*^*2*^ = 0%).

#### Depression in taste dysfunction compared to the control group

**Fig 6:**
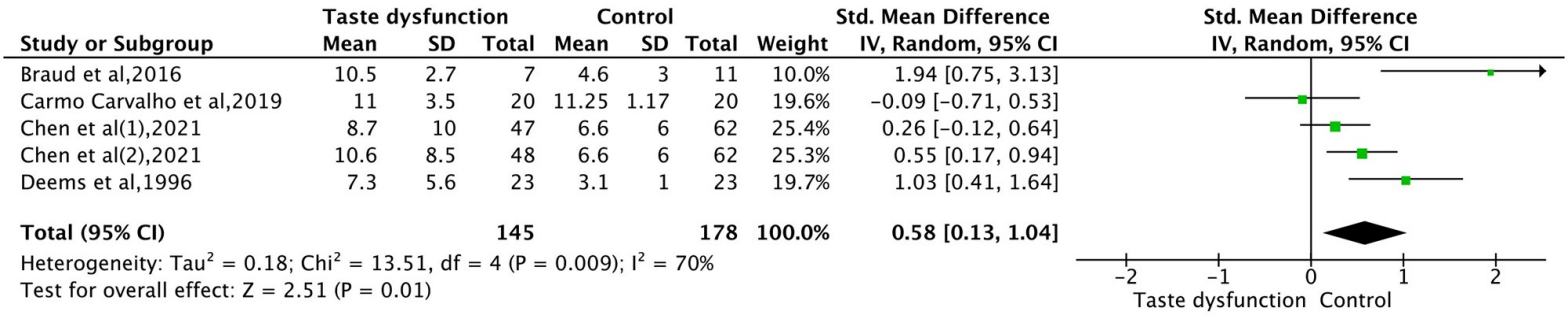
Forest plot of changes in depression scale score between gustatory dysfunction patients and controls in Observational Studies. In Chen’s study, data was isolated based on gustatory dysfunctions only, and mixed gustatory and olfactory dysfunctions were calculated with mean and standard error. Hence, their raw data was adopted; Chen Ben(1) represents gustatory dysfunctions data, and Chen Ben(2) represents mixed gustatory and olfactory dysfunctions data.

Evidence from Observational Studies. To compare depressive disorders between gustatory dysfunction patients and controls, a total of four articles with 261 participants were included. Fig 6 revealed that patients with taste dysfunction are significantly more likely to develop depression than those without (*p* = 0.01, 95% CI: 0.58 [0.13, 1.04]). However, there is a slightly higher heterogeneity among the five data items, and a random effects model was employed (*p* = 0.009, *I*^*2*^ = 70%). Sensitivity analysis indicated that the association between taste disorders and depression was not noticeably affected by an individual literature.

**Fig 7:**
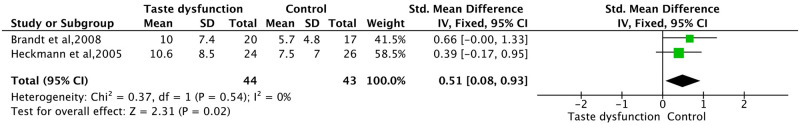
Forest plot of changes in depression scale score between gustatory dysfunction patients and controls in randomized trials.

Evidence from randomized trials is shown in Fig 7. A total of two articles with 87 participants were included. The analysis revealed that patients with taste dysfunction are significantly more likely to develop depression than those without (*p* = 0.02, 95% CI: 0.51 [0.08, 0.93]), and no heterogeneity was observed (*p* = 0.54, *I*^*2*^ = 0%).

### Sensitivity analysis and publication bias

**Fig 8:**
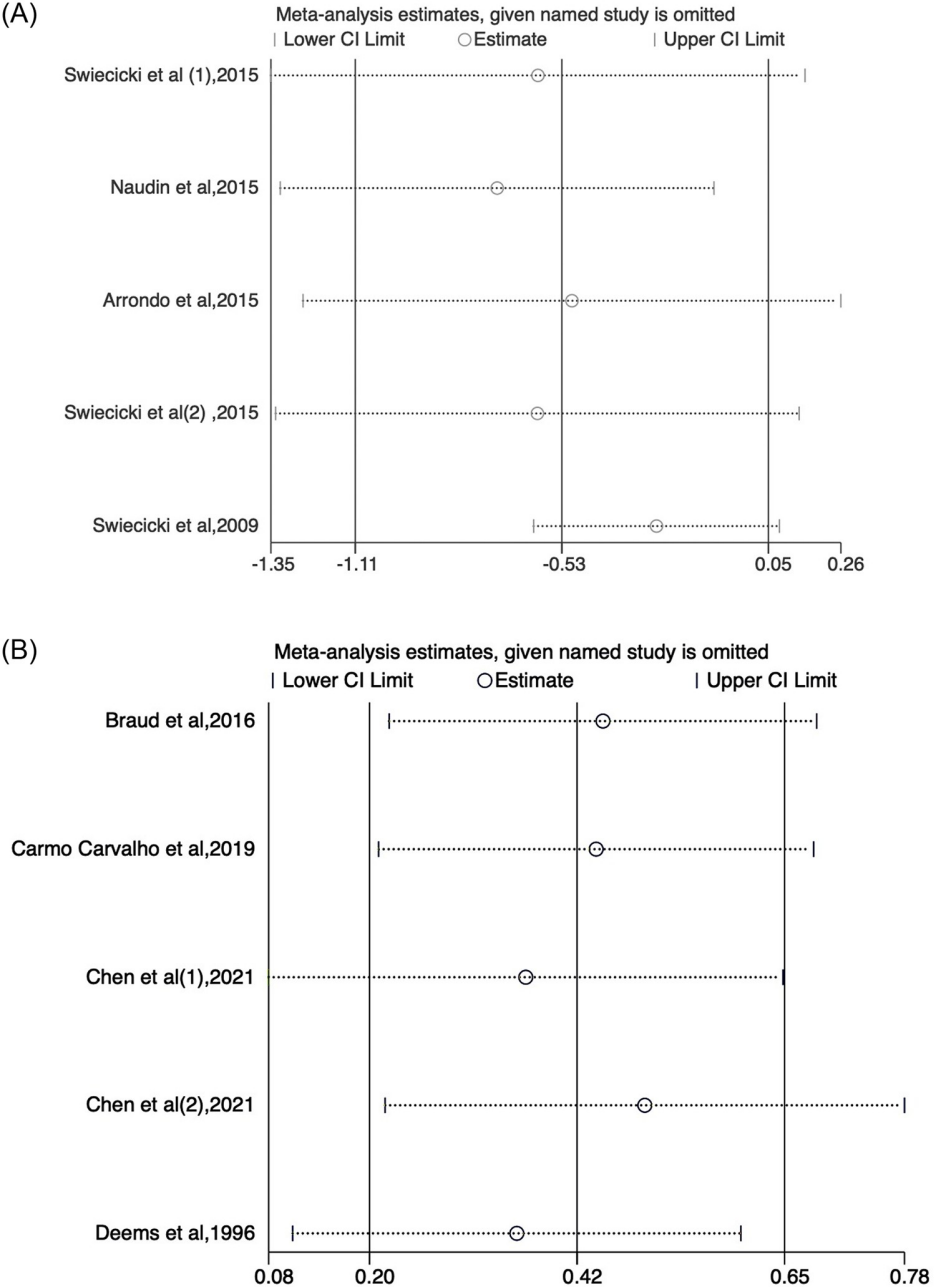
Sensitivity analysis. A: Sensitivity analysis for the pleasantness ratings of sucrose solutions (hedonic valuation) in depressed patients versus controls. Sensitivity analyses showed the robustness of the outcomes, with no single study unduly influencing the analyses. B: Sensitivity analysis for depression between taste dysfunction and control in observational studies. The two ends of the lines represent the 95% CI.

A sensitivity analysis was conducted based on the meta-analysis, examining the hedonic value and depression scores of individuals with gustatory dysfunction using the leave-one-out approach. The exclusion of individual studies did not alter the results, and there were no significant differences in the direction and magnitude of the combined estimation. This suggests that no publication bias resulting from individual studies was detected. Therefore, the meta-analysis outcomes were deemed robust and reliable (Fig 8A and 8B).

**Fig 9:**
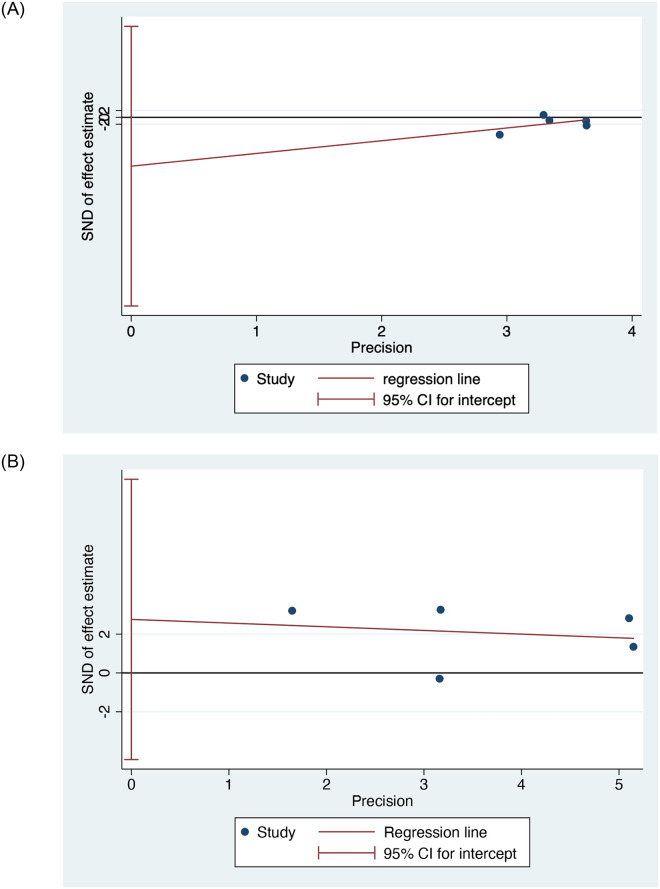
Egger’s test. A: The hedonic value of the depressed group and controls. B: The depression between gustatory dysfunction and control.

Additionally, Egger’s test was conducted to assess publication bias. The results indicated that there was no significant publication bias for the hedonic value comparison between depressed patients and controls (Fig 9A) (*p* = 0.347), as well as for the comparison of depression in groups with and without taste disorders (Fig 9B) (*p* = 0.311). Both tests from Egger’s test yielded *p* values greater than 0.05, suggesting the absence of publication bias.

### Grading the quality of evidence

The quality of evidence was judged as moderate that the correlation between taste and depression in patients with taste disorders, which was downgraded from high due to inconsistency of results.

## Discussion

### Gustatory function changes in the literature

This research primarily aims to analyse the differences in gustatory function between depressed patients and controls. Due to a lack of understanding regarding the association between gustatory function and depression, various measures of gustatory function have emerged in the literature. Previous studies have also presented divergent results regarding gustatory function.

By comparing ten studies that met the criteria, it was found that three studies [76, 84, 86] demonstrated significant differences in certain aspects of gustatory function between depressed and non-depressed controls. The depression scores also reflected the level of gustatory function. However, the remaining seven studies [6, 26, 27, 81–83, 87] showed no significant differences between the two groups. To fully understand the relationship between gustatory function and depression, it is crucial to comprehensively analyse the literature and interpret the research results. After a more cautious interpretation of the results, it was found that although some results were not significant, in certain studies, the average performance of the control group was better than that of the depression group [6, 27, 81]. The most consistent difference in gustatory function lies in the perception threshold of sweet taste, with analysis showing that the ability of depression patients to recognize sweet taste is significantly lower than that of controls [41, 84]. Among the four studies [27, 81, 83, 87] that measured gustatory identification ability (also known as taste sensitivity), only one study [81] found significant differences between the two groups.

Furthermore, the pleasure ratings of sucrose solutions (hedonic evaluation) are common topics in gustatory function, covered by seven studies [26, 43, 76, 82–84, 87]. Although more than half of the literature showed that the control group performed better than the diseased group, these studies did not find significant differences between the depression group and the control group, and the research conclusions were inconsistent. Perhaps a larger sample size study may yield more consistent outcomes.

Overall, there are disagreements in current research regarding the relationship between depression and gustatory function, with only a few studies supporting alterations in gustatory function among patients with depression. Depression might impact various aspects of gustatory function [76]. However, additional animal and clinical studies are necessary to elucidate the underlying mechanisms, including evidence from proteomics and metabonomics, neurochemistry, genetics, and neuroimaging. It is important to note that individuals may experience varying degrees of impact and different types of depression may manifest differently. Other related comorbidities and drug use could also contribute, potentially leading to adverse effects on taste recognition and processing.

### Depressive symptoms changes in the literature

This part of the study aims to determine whether individuals with gustatory dysfunction are more prone to experiencing depression. One study [39] indicated that most patients with chemical sensory disorders exhibited mild depressive symptoms. The depression scores were higher in individuals with mixed olfactory and gustatory dysfunctions than those with olfactory dysfunctions and controls. However, the depression scores of patients with gustatory dysfunction alone did not differ greatly from those of the controls. In the research conducted by [40, 44], it was observed that the depression scores of patients with gustatory dysfunction were significantly higher than those of the controls.

Additionally, a significant association was found between the number of symptoms related to gustatory function and the depression scores (*r* = 0.501, *p* < 0.05) [44]. Another study [42] suggested that patients with gustatory dysfunction who exhibit lower levels of depression are more likely to recover gustatory function. Two studies [35, 41] discovered that improvements in gustatory function and reductions in psychological symptoms occurred simultaneously, with significant differences observed when compared to the control group.

In conclusion, the analysis reveals that individuals with taste dysfunction frequently exhibit symptoms of depression and have a higher likelihood of experiencing depression. Psychological factors can influence the patient’s perception of gustatory function when gustatory dysfunction is present as an underlying condition. This explains why some patients solely express dissatisfaction with the absence of taste in food, while others report experiencing additional distress [88].

### Limitations in the literature

This integrated review and meta-analysis exploring the correlation between depression and gustatory function have certain limitations. First, the included studies varied regarding disease characteristics, participant demographics, evaluation tools, and operational methods of gustatory function. These differences may introduce heterogeneity and bias into the analysis. As noted in previous studies [5], literature is scarce on gustatory function, along with a lack of objective evaluation tools. Secondly, most of the research primarily focuses on test methods and essential data regarding gustatory function, while the sensory perception of depressed patients receives less attention.

Moreover, some studies have included heterogeneous populations, encompassing individuals with depression (unipolar and bipolar disorder) or psychosis (seasonal depression and borderline personality disorder), which makes it challenging to extract specific data pertaining to individuals with depression. Therefore, the findings of this integrated review should be interpreted with caution. Lastly, excluding only Chinese literature may limit the retrieval of relevant Chinese papers but may not cover the complete spectrum of available research.

## Conclusions and significance for future research

In this systematic review, we conducted a comprehensive literature search and summarized the relationship between depression and gustatory function. Our findings suggest that patients with depression exhibit abnormalities in certain aspects of gustatory function in the perception threshold of sweet taste and the pleasure ratings of sucrose solutions (hedonic evaluation), and individuals with abnormal gustatory function may be more prone to experiencing depressive symptoms, as mentioned earlier, there is a significant correlation between abnormal taste and depression scores, and patients with lower levels of depression are more likely to recover taste function, with significant improvements in both taste and mood. However, the linear correlation between depression and gustatory function is not statistically significant, which could be attributed to the choice of taste testing methods. Most studies employed relatively crude taste testing methods that may not comprehensively reflect the patient’s gustatory function. Qualitative gustatory function tests demonstrate gustatory dysfunction, whereas quantitative gustatory tests rarely capture this situation. Therefore, future research should employ more detailed and targeted gustatory testing to further elucidate the relationship between depression and gustatory function. Additionally, it remains uncertain whether there are clinically significant differences among individual patients. It is unclear whether the coexistence of depression and gustatory dysfunction results in worse health outcomes compared to these two conditions independently. In summary, the relationship between depression and taste is effected by multiple aspects, and more high quality studies are needed to demonstrate effectiveness.

The advantage of this study is that our study included 17 studies involving a large number of participants, which improved the ability to discover significant associations and provided more reliable estimates. In addition, we also conducted a comprehensive evaluation of taste function through various testing indicators, avoiding relying solely on a single measurement method and providing a more comprehensive understanding of taste function and depression.

In our research, several innovative points should be mentioned. Firstly, we conducted our first study on the relationship between depression and taste perception. Previous studies have only reviewed the relationship between smell and depression, or the relationship between bidirectional affective disorder and taste, without conducting a comprehensive analysis of taste and depression. Secondly, the included studies were designed as randomized and case-control studies, where recall and selection bias can be significantly reduced. In addition, sensitivity analysis shows that excluding any single study has little impact on overall risk estimation. All of these measures support the robustness of the research results.

Future research should prioritize investigating whether taste dysfunction can serve as an effective tool for early screening of depression. Additionally, it would be valuable to explore critical factors such as the prevalence of taste dysfunction among depressed populations and the prevalence of depression within the gustatory dysfunction population. Examining the impact of different types of depression on gustatory function and assessing how various causes of taste dysfunction influence depressive symptoms are also important considerations for future studies. Furthermore, factors such as hormone levels, disease aetiology, disease progression, and medication parameters of the participants should be taken into account. However, obtaining accurate data on these influencing factors can be challenging in practical clinical settings, and long-term follow-up and treatment monitoring can be difficult to achieve. Therefore, one feasible option for future research is to establish relevant animal models that evaluate sensory functions related to depressive-like behaviour [83] and subsequently conduct clinical population retesting based on successful outcomes. Additionally, it is crucial to standardize gustatory function testing methods and processes to ensure uniformity across studies. In summary, further research endeavors will provide deeper insights into the neurobiological underpinnings of depression.

## Supporting information

S1 Checklist (PDF)

S1 File Supplementary material: Search strategies. (PDF)

S2 File PRISMA 2015 checklist. (PDF)

S3 File Funding. (PDF)

S4 File Supplementary material: Quality of the evidence (GRADE) for RCTs. (PDF)
