# Supplementary material for: Taste dysfunction as a predictor of depression in schizophrenia: A systematic review and meta-analysis
Source: PLoS One. 2024 Mar 22;19(3):e0300935. doi: 10.1371/journal.pone.0300935 (PMC10959346; doi:10.1371/journal.pone.0300935)
Supplement: S1 File — (PDF) [file pone.0300935.s002.pdf]

S1 Appendix A. Search terms

Taste dysfunction	Depression
International database	
Taste dysfunction; Taste Disorder; Gustatory dysfunction; Taste disorder; Sense organ disorder; Dysgeusia; Dysgustatory; Gustatory function; Gustation; Taste; Taste Sense; Taste Disorder, Primary, Sweet; Taste Disorder, Secondary, Bitter; Taste Disorder, Secondary, Salt; Taste Disorder, Secondary, Sweet;	Depression, Depressive disorder; Major depression; Depressive Disorder; Major, Depressive Symptoms; Depressive Syndrome; Emotional Depression; Depression, Neurotic; depressed disorder; Endogenous Depressions; Symptoms, Depressive; Unipolar Depression
Chinese database	
“味觉障碍”、“味觉异常”、“感觉功能异常”、“口味异常”、“味觉失灵”、“感官障碍”、“感觉”、“口味”、“味觉”	“抑郁症”、“抑郁”、“郁证”、“重度抑郁”、“抑郁症状”、“抑 郁情绪”、“抑郁量表”

S1 Appendix B. Search strategy in PubMed

1	((((((((((((((((Taste dysfunction) OR (Taste Disorder)) OR (Taste Disorder, Anterior Tongue)) OR (Taste Disorder, Primary)) OR (Primary Taste Disorder)) OR (Taste Disorder, Primary, Bitter)) OR (Taste, Metallic)) OR (Metallic Tastes)) OR (Taste Disorder, Primary, Sweet)) OR (Taste Disorder, Secondary)) OR (Secondary Taste Disorder)) OR (Taste Disorders, Secondary)) OR (Taste Disorder, Secondary, Bitter)) OR (Taste Disorder, Secondary, Salt)) OR (Taste Disorder, Secondary, Sweet)) OR (Taste Dysfunction)) OR (Dysfunction, Taste)) OR (Taste Disorder, Primary, Salt)
2	(((((((((((((((((((((((Depressive Disorders) OR (Disorder, Depressive)) OR (Disorders, Depressive)) OR (Neurosis, Depressive)) OR (Depressive Neuroses)) OR (Depressive Neurosis)) OR (Neuroses, Depressive)) OR (Depression, Endogenous)) OR (Depressions, Endogenous)) OR (Endogenous Depression)) OR (Endogenous Depressions)) OR (Depressive Syndrome)) OR (Depressive Syndromes)) OR (Syndrome, Depressive)) OR (Syndromes, Depressive)) OR (Depression, Neurotic)) OR (Depressions, Neurotic)) OR (Neurotic Depression)) OR (Neurotic Depressions)) OR (Melancholia)) OR (Melancholias)) OR (Unipolar Depression)) OR (Depression, Unipolar)) OR (Depressions, Unipolar)) OR (Unipolar Depressions)
3	1 AND 2

S1 Appendix C. Search strategy in Embase

#1	'depression' OR 'depressive symptoms' OR 'depressive symptom' OR 'symptom, depressive' OR 'symptoms, depressive' OR 'emotional depression' OR 'depression, emotional' OR 'anxiety disorder' OR 'major depression'
#2	'taste' OR 'taste disorder' 'taste dysfunction' OR 'xerostomia' OR 'paresthesia' OR 'mental disease'' OR 'taste disorders'
#3	#1 AND #2
#4	#3 AND 'human'/de AND 'controlled study'/de

S1 Appendix D. Search strategy in Cochrane Library

#1	MeSH descriptor: [Depression] explode all trees
#2	Depressive Symptoms
#3	Depressive Symptom
#5	Symptoms, Depressive
#6	Depression, Emotional
#7	Emotional Depression
#8	major depression
#9	#1 or #2 or #3 or #4 or #5 or #6 or #7 or #8
#10	MeSH descriptor: [Taste] explode all trees
#11	Tastes
#12	Taste Sense
#13	Gustations
#14	Taste Senses
#15	Senses, Taste
#16	#10 or #11 or #12 or #13 or #14 or #15
#17	#9 and #16

S1 Appendix E. Search strategy in Ovid Medline

"taste disorders"OR "taste disorder" OR "taste disorder, primary" OR "dysgeusia" OR "gustation" AND "depression" OR "depressive Symptoms" OR "major depression"

S1 Appendix F. Search strategy in EBSCO

(MH _Depressive Disorder, Major_) OR (MH _Depressive Disorder_) AND (MH _Taste_) OR (MH _Gustation_)

S1 Appendix G. Search strategy in Web of Science

Depression OR Depressive disorder OR Major depression AND Taste dysfunction OR Taste Disorder OR Gustatory dysfunction OR Dysgeusia

S1 Appendix H. Search strategy in CNKI

(((主题=中英文扩展(depression) 或者 题名=中英文扩展(major, depressive Symptoms) 或者 v_subject=depression 或者 title=major, Depressive Symptoms) 并且 (主题=中英文扩展(taste) 或者 题名=中英文扩展(taste) 或者 v_subject=taste 或者 title=taste dysfunction)) 或者 ((主题=中英文扩展(major depression) 或者 题名=中英文扩展(major depression) 或者 v_subject=major depression 或者 title=major depression) 并且 (主题=中英文扩展(dysgeusia) 或者 题名=中英文扩展(gustatory dysfunction) 或者 v_subject=taste dysfunction 或者 title=sense organ disorder))) (模糊匹配)

S1 Appendix I. Search strategy in Grey Literature

Depression OR Depressive Symptoms OR Emotional Depression OR Depressive Disorder AND taste OR tastes OR Taste Sense OR Gustation
