# Supplementary material for: Taste dysfunction as a predictor of depression in schizophrenia: A systematic review and meta-analysis
Source: PLoS One. 2024 Mar 22;19(3):e0300935. doi: 10.1371/journal.pone.0300935 (PMC10959346; doi:10.1371/journal.pone.0300935)
Supplement: S3 File — (PDF) [file pone.0300935.s004.pdf]

Funding

1. This research was supported by State Administration of Traditional Chinese Medicine of the People's Republic of China Funding Project: Pei Lin's National Prestigious Chinese Physicians Inheritance Studio(State TCM Human Education Letter [2022] No. 75), provided computer and related software for this work, and Lin Pei is the recipient of the funding awards. He is responsible for writing – review & editing work.

2. Hebei Province Graduate Innovation Funding Project (grant No. XCXZZBS2023011), provided training funding for the meta-analysis, and Jia Liu is the recipient of the funding awards. She is responsible for formal analysis, writing – original draft work.

3. Hebei Provincial Administration of Traditional Chinese Medicine Funding Project (grant No. 2022113), and Jia Liu is the recipient of the funding awards, provided databases search costs.

4. Hebei Provincial Administration of Traditional Chinese Medicine Funding Project (grant No. 2022112), Xin Ping is the recipient of the funding awards and provided language polishing costs. He is responsible for software operation work.
