# Supplementary material for: Taste dysfunction as a predictor of depression in schizophrenia: A systematic review and meta-analysis
Source: PLoS One. 2024 Mar 22;19(3):e0300935. doi: 10.1371/journal.pone.0300935 (PMC10959346; doi:10.1371/journal.pone.0300935)
Supplement: S4 File — (PDF) [file pone.0300935.s005.pdf]

Grading of Recommendations, Assessments, Developments and Evaluations (GRADE) approach for certainty in evidence

Quality assessment							No of patients		Effect		Quality	Importance
**No of studies**	**Design**	**Risk of bias**	**Inconsistency**	**Indirectness**	**Imprecision**	**Other considerations**	**Changes in depression scale score between gustatory dysfunction patients and controls**	**Control**	**Relative (95% CI)**	**Absolute**		
**RCTs (Better indicated by lower values)**												
2	Randomized trials	No serious risk of bias	Serious	No serious indirectness	No serious imprecision	None	44	43	-	SMD 0.51 higher (0.08 to 0.93 higher)	⊕⊕⊕◯ Moderate	Critical

GRADE Working Group grades of evidence

High quality: Further research is very unlikely to change our confidence in the estimate of effect.

Moderate quality: Further research is likely to have an important impact on our confidence in the estimate of effect and may change the estimate.

Low quality: Further research is very likely to have an important impact on our confidence in the estimate of effect and is likely to change the estimate.

Very low quality: We are very uncertain about the estimate.
